# Modelling Aotearoa New Zealand's COVID-19 protection framework and the transition away from the elimination strategy

**DOI:** 10.1098/rsos.220766

**Published:** 2023-02-01

**Authors:** Giorgia Vattiato, Audrey Lustig, Oliver Maclaren, Rachelle N. Binny, Shaun C. Hendy, Emily Harvey, Dion O'Neale, Michael J. Plank

**Affiliations:** ^1^ School of Mathematics and Statistics, University of Canterbury, Christchurch, New Zealand; ^2^ Department of Physics, University of Auckland, Auckland, New Zealand; ^3^ Te Pūnaha Matatini, Auckland, New Zealand; ^4^ Manaaki Whenua, Lincoln, New Zealand; ^5^ Department of Engineering Science, University of Auckland, Auckland, New Zealand; ^6^ M.E. Research, Takapuna, Auckland, New Zealand

**Keywords:** COVID-19, Delta, public health, border restrictions, vaccination, lockdown

## Abstract

For the first 18 months of the COVID-19 pandemic, New Zealand used an elimination strategy to suppress community transmission of SARS-CoV-2 to zero or very low levels. In late 2021, high vaccine coverage enabled the country to transition away from the elimination strategy to a mitigation strategy. However, given negligible levels of immunity from prior infection, this required careful planning and an effective public health response to avoid uncontrolled outbreaks and unmanageable health impacts. Here, we develop an age-structured model for the Delta variant of SARS-CoV-2 including the effects of vaccination, case isolation, contact tracing, border controls and population-wide control measures. We use this model to investigate how epidemic trajectories may respond to different control strategies, and to explore trade-offs between restrictions in the community and restrictions at the border. We find that a low case tolerance strategy, with a quick change to stricter public health measures in response to increasing cases, reduced the health burden by a factor of three relative to a high tolerance strategy, but almost tripled the time spent in national lockdowns. Increasing the number of border arrivals was found to have a negligible effect on health burden once high vaccination rates were achieved and community transmission was widespread.

## 1. Introduction

Since the beginning of the COVID-19 pandemic in 2020, New Zealand has followed an elimination strategy that involved minimizing community transmission of the virus until high vaccine coverage was reached. This was achieved with a combination of strict border restrictions to minimize the number of imported cases, and a rapid public health response to eliminate border-related incursions. Until November 2021, this involved a four-tier alert level system ranging from alert level 1 (few or no restrictions on domestic activities) to alert level 4 (stay-at-home orders and closure of schools and non-essential business) These alert levels were applied nationally and regionally to successfully eliminate three COVID-19 community outbreaks: March–May 2020, August–September 2020 and February–March 2021.

On 17 August 2021, a case of COVID-19, later confirmed to be the B.1.617.2 (Delta) variant of SARS-CoV-2, was detected in the community with no clear link to the border. At the time, New Zealand's vaccine roll-out was still in its early stages and the population had very low levels of immunity: only 38% of the eligible population had received at least one vaccine dose and there had only been approximately 3000 confirmed cases of COVID-19, approximately 0.06% of the population. Despite a rapid and effective public health response, the outbreak was not eliminated. Instead, it was suppressed with a mixture of non-pharmaceutical interventions and continued border restrictions as vaccine coverage increased to high levels.

In the following four months, the outbreak caused a total of approximately 9000 cases, largely contained to the Auckland region, while rapid progress was made with the vaccine roll-out. By early December 2021, 94% of the eligible population (12+ years old) had received at least one dose of the Pfizer-BioNTech BNT162b2 vaccine and 90% of the eligible population had received two doses. This enabled New Zealand to formally move away from the elimination strategy to a mitigation strategy.

As part of this move, the alert level system was replaced with a new COVID-19 Protection Framework [1] on 3 December 2021. This new system comprised three different ‘traffic lights’ or levels of restrictions (red, orange and green; red having the highest restrictions). It also included a comprehensive vaccine pass system, wherein vaccination was required for workers in healthcare, education and other public sector organizations, and for entry to many non-essential business, services and events. The aim of the new system was to control transmission of the virus and minimize health impacts while allowing more freedoms and avoiding national lockdowns. Auckland and most of the upper and central North Island started at the ‘red’ setting. The lower North Island and the South island started at ‘orange’.

Since the early stages of the pandemic, New Zealand has operated under a strict border control strategy, only allowing a limited number of New Zealand residents or citizens to enter the country, with a mandatory 14-day stay in a managed isolation and quarantine facility on arrival. In August 2021, the New Zealand Government has announced a plan for relaxing border restrictions from the first quarter of 2022, following completion of the primary vaccine roll-out. This consisted of a phased approach, with quarantine requirements eased first on New Zealand citizens and residents before expanding to other travellers, and the creation of low-, medium- and high-risk pathways depending on the traveller's country of origin and vaccination status.

Following the emergence of the more transmissible Delta variant in 2021, it became clear that even very high levels of vaccine coverage would probably not, on their own, be sufficient to reach population immunity (i.e. a state where the effective reproduction number is less than 1 in the absence of any other control measures) [2]. As a result, other public health interventions would be required alongside vaccination to control the virus and minimize its health impacts.

This paper describes a modelling study carried out in the second half of 2021 to understand potential epidemic trajectories of the Delta variant in the New Zealand, their health impact, and how they may respond to different management strategies and border reopening plans. Alongside a model of the risk of community outbreaks under different border measures and different levels of vaccine coverage [3], the results of this work were used to inform government strategy, reopening plans and outbreak response. In particular, the model allows government officials and decision makers to compare strategies that aim to suppress the virus to low levels and those that allow higher levels of community transmission. It also allows trade-offs between restrictions in the community and restrictions at the border to be explored. This work pre-dates the emergence of the Omicron variant [4,5] and its introduction to New Zealand, which has necessitated a change in strategy.

In this report, we investigate how infection, number of detected cases, hospital admissions, death and time spent under different traffic light settings vary with different combinations of vaccine effectiveness, vaccine coverage, numbers of quarantine-free arrivals, and additional public health and social measures. In particular, we investigate the effect of three different outbreak management responses (low, medium and high tolerance response) that ease and tighten restrictions based on case numbers and hospitalizations. We use a stochastic age-structured transmission model for the Delta variant that builds on a previous model of New Zealand's vaccine roll-out [2]. The model incorporates the effects of vaccination, age structure, case isolation, contact tracing and control measures.

## 2. Methods

### 2.1. Age-structured stochastic model of SARS-CoV-2 transmission and control

We use an age-structured stochastic model for transmission of SARS-CoV-2 in a partially vaccinated population, parametrized to represent New Zealand's age structure and age-specific contact rates [2]. We consider a basic reproduction number *R*_0_ = 6.0 and a generation interval with mean = 5.05 days and s.d. = 1.9 days, approximately representing the Delta variant of SARS-CoV-2 [6]. This model distinguishes between clinical and subclinical (asymptomatic) infections, the latter having a 50% infectiousness reduction [7,8]. We assume age-stratified hospitalization and fatality rates based on the increased severity of the Delta variant (electronic supplementary material, table S3). We also include the effects of vaccination and case-targeted controls (case isolation and contact tracing). The vaccine coverage is fixed at the beginning of the simulations and we do not model concurrent vaccination and transmission. This describes a situation where controls aimed at suppressing the virus are relaxed after a given level of vaccine coverage is reached. While there is some uncertainty in vaccine effectiveness, and effectiveness is known to wane with time, our baseline assumptions are chosen to best represent the current understanding of the effect of two doses of the Pfizer/BioNTech BNT162b2 vaccine on the Delta variant. For a complete model specification, see the electronic supplementary material.

### 2.2. Vaccination coverage and effectiveness

At the time this modelling was undertaken, the roll-out of booster doses in New Zealand had not started. Although a booster programme was planned, in the pre-Omicron era this was primarily envisaged as counteracting the effects of waning immunity after two doses. As a model simplification, we ignore the dynamic effects of waning and boosting, and assume that vaccination provides a fixed level of protection at the population level by using constant vaccine coverage and vaccine effectiveness parameters. We assume a static vaccination coverage, corresponding to the first dose vaccination coverage seen in New Zealand as of 3 November 2021, scaled up to obtain a 90% national coverage (electronic supplementary material, table S3). The model does not make the distinction between one or two vaccination doses and assumes everyone is either unvaccinated or double-vaccinated.

### 2.3. Public health measures and response strategy

The model includes a time-varying control function to simulate the effects of different public health measures that may be eased or tightened in response to case numbers and/or hospitalizations. Baseline public health measures and mitigation are in place and assumed to provide a 10% reduction in transmission under a green setting, a 20% reduction under the orange setting, a 30% reduction under the red setting, and a 60% reduction under the emergency setting, representing strict stay-at-home orders and business closures. These numbers are broadly in line with international estimates, for example, those by [9] who estimated a possible relative reduction in *R* of 0–20% from non-pharmaceutical interventions not including lockdowns, and of 75–87% from lockdowns. The emergency setting reduction is in line with estimates of the control function during alert level 3 in October 2021 [10]. Simulations start in the red setting (30% reduction in transmission) and stay in red for at least the first 28 days. We investigate the effect of different triggers for raising/lowering the traffic light setting based on case numbers and number of hospitalizations, representing low-, medium- and high-tolerance scenarios (table 1). The low- and medium-tolerance scenarios use case numbers as triggers to raise and lower the traffic light setting, whereas the high-tolerance scenario ignores case numbers and only uses the number of hospital beds currently occupied as trigger. We also investigate a ‘very low’-tolerance scenario in our sensitivity analysis of border and community seed cases to better understand the impact of different border settings.

**Table 1.** Trigger criteria used to raise/lower traffic light settings (G = green, O = orange, R = red, E = emergency) for low-, medium- and high-tolerance outbreak management responses. The trigger number of cases refers to the average number of new daily cases over the last 7 days. The trigger number of hospital beds corresponds to the current number of hospital beds occupied with COVID-19 patients. The low- and medium-tolerance scenarios respond to case numbers; the high-tolerance scenario responds to hospitalizations.

			very low tolerance^a^	low tolerance	medium tolerance	high tolerance
escalation criteria	→ O	cases	10	50	200	—
		hosp beds	—	—	—	100
	→ R	cases	25	100	400	—
		hosp beds	—	—	—	200
	→ E	cases	50	300	1200	—
		hosp beds	—	—	—	600
relaxation criteria	→ G	cases	0	0	100	—
		hosp beds	—	—	—	50
	→ O	cases	10	75	300	—
		hosp beds	—	—	—	150
	→ R	cases	30	200	800	—
		hosp beds	—	—	—	400

^a^The ‘very low tolerance’ triggers were only used for the sensitivity analysis of border and community seeds.

### 2.4. Test-trace-isolate-quarantine

The model also includes a test-trace-isolate-quarantine (TTIQ) system (described in the electronic supplementary material). The effect of this system varies depending on our assumptions about testing probability, proportion of contacts traced and effectiveness of quarantine and isolation (electronic supplementary material, table S2). We assume 45% of clinical and 0% of subclinical cases are detected via testing. Detection occurs with an exponentially distributed delay from symptom onset with mean 4 days. Once an infection is detected, the individual is assumed to be immediately isolated, resulting in an 80% transmission reduction. Some transmission may still happen within the household and isolation compliance is not perfect, hence we do not model isolation as 100% effective in reducing onward transmission. Contact tracing parameters are dependent on the number of daily cases. If the number of daily detected cases remain on average below 100 cases (contact tracing capacity) for 12 consecutive days, a proportion *p*_trace_ = 0.7 of secondary infections of a confirmed case are identified via contact tracing and quarantined. Quarantine occurs with a gamma-distributed delay from detection of the index case with a mean of 3 days and a s.d. of 1.7 days. If the number of daily detected cases exceed the contact tracing capacity, no secondary infections can be identified and quarantined. Traced contacts are assumed to have a 50% transmission reduction from quarantine.

### 2.5. Initial conditions

Simulations are initialized with *N*_0_ = 2000 initial cases, seeded in the community in the first 14 days of the simulated time period. The seed cases' age distribution and vaccination status are representative of the population's age distribution and vaccination coverage as of 27 October 2021 (electronic supplementary material, table S3). In addition, *N*_border_ = 5000 infections per year are regularly seeded into the community during the simulated time period, representing infected travellers arriving into the New Zealand community. We investigate scenarios with different values of *N*_border_, representing different levels of travel volume and border restrictions, as described in table 2. Each border seed case is assumed to have received two doses of the vaccine and to have a further 80% transmission reduction, representing testing and isolation requirements for travellers [3].

**Table 2.** Parameters used in the scenario analysis.

parameters	baseline values	scenarios tested
number of initial seed cases in the community^a^	2000	0, 500, 1000, 2000, 10 000
number of infections in border arrivals (per year)	5000	0, 500, 5000, 10 000, 20 000
border cases isolation effectiveness (%)	80 (12 days quarantine, test on day 3 and 12)	20 (7 days home isolation)

^a^Initial infections in the community seeded in the first 14 days of the simulated period.

### 2.6. Scenario analysis

In addition to the baseline scenarios, we explore the effects of changing model assumptions for the initial number of cases, the number of border cases and the effectiveness of border case isolation (table 2). In the electronic supplementary material of this paper we also explore the effect of varying community case isolation, the probability of case detection, the effectiveness of control measures, vaccine effectiveness, the capacity of the contact tracing system and the risk of hospitalization (electronic supplementary material, table S5).

From each simulation, we output the number of infections, detected cases, hospital admissions and deaths, and the time spent under different traffic light settings. All simulations were run for a 1-year period and results were averaged over 50 independent simulations of the stochastic model for each set of parameters.

## 3. Results

### 3.1. Baseline scenario

**Figure 1.**
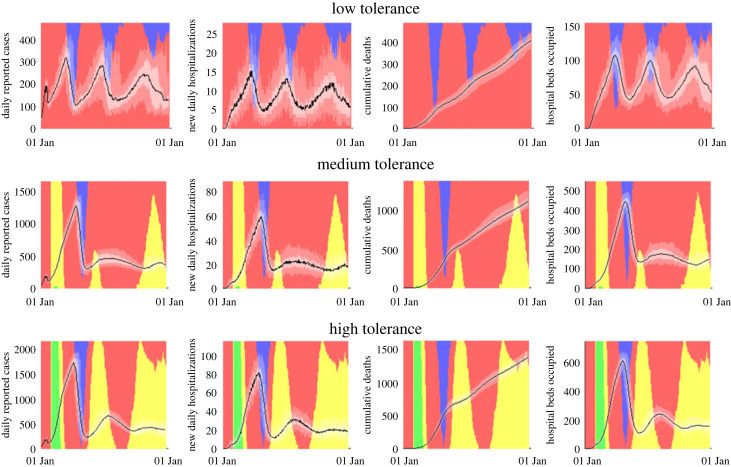
Time series of daily reported cases, new daily hospitalizations, cumulative deaths and hospital beds occupied for the low-, medium- and high-tolerance strategies under the baseline model scenario. The black curves represent the median of *N* = 70 simulations and shaded areas represent 50% and 95% percentile ranges. Background colours represent the proportion of simulations that were in each traffic light setting (green, yellow, red, emergency: blue) at a given moment in time. Note that the *y*-axes are different for each scenario.

In the baseline scenario and with a medium tolerance response, there are approximately 165 000 cases, 7600 hospitalizations and 1100 deaths during a 1-year simulation of the model. The peak number of hospital beds occupied is 470. 72% of the time (approx. 9 months) is in the red traffic light setting and 5% of the time (approx. 18 days) is in the emergency setting (table 3, figure 1). A low tolerance response leads to approximately a threefold reduction in hospitalizations and deaths but triple the time spent in the emergency setting. The first row in figure 1 shows three distinct periods during the simulated year when the emergency setting was triggered by increasing case numbers. The three ‘emergency’ periods were usually short, each spanning approximately one month in most simulations, but they resulted in very low peak outcomes for cases and hospitalizations. A high-tolerance response leads to approximately a 25% increase in hospitalizations and 35% increase in deaths relative to medium tolerance, with a similar amount of the time under the emergency setting. This is due to the difference in the relaxation threshold, which is measured in number of hospital beds for the high-tolerance scenario and in number of cases in the medium-tolerance scenario (electronic supplementary material, table S4). Both the escalation and relaxation triggers of the high-tolerance scenario are higher than those of the medium-tolerance strategy, which means that the emergency setting is both entered and left at higher case and hospital beds numbers than in the medium-tolerance scenario (figure 1, row 3).

**Table 3.** Median number of infections, detected cases, hospitalizations, peak hospital occupancy and deaths over a year under the low-, medium- and high-tolerance outbreak management response for all scenarios tested. The G, O, R and E columns indicate the median percentage time spent in each of the traffic light setting (green, orange, red and emergency). The TTIQeff indicate the reduction in transmission as a result of the test, trace, isolate, quarantine system.

scenario	infections	cases	hospitalizations	peak beds	deaths	G (%)	O (%)	R (%)	E (%)	TTIQeff (%)
*baseline scenario*										
very low tolerance	32 000	13 000	400	40	50	0	3	82	15	12
low tolerance	215 000	66 000	2900	130	410	0	0	85	15	8
medium tolerance	553 000	165 000	7600	470	1100	0	23	72	5	8
high tolerance	684 000	204 000	9500	650	1390	7	38	49	6	8
*A1. border cases = 10K*										
very low tolerance	37 000	15 000	400	40	50	0	0	78	22	12
low tolerance	217 000	66 000	2900	130	400	0	0	84	16	8
medium tolerance	551 000	165 000	7500	470	1090	0	21	75	5	8
high tolerance	690 000	206 000	9500	650	1390	6	38	50	6	8
*A2. border cases = 20K*										
very low tolerance	46 000	17 000	400	40	50	0	0	61	39	11
low tolerance	223 000	67 000	2800	130	400	0	0	84	16	8
medium tolerance	554 000	166 000	7400	460	1080	0	19	76	5	8
high tolerance	703 000	210 000	9600	650	1410	6	38	50	6	8
*A3. community seed cases = 10 K, border cases = 10K*										
very low tolerance	93 000	32 000	1100	200	150	0	0	75	25	10
low tolerance	245 000	75 000	3200	210	450	0	0	82	18	8
medium tolerance	588 000	176 000	8000	470	1170	0	12	83	5	8
high tolerance	725 000	217 000	9900	640	1460	0	43	51	6	8
*A4. community seed cases = 10 K, border cases 20K*										
very low tolerance	103 000	34 000	1100	210	150	0	0	60	40	9
low tolerance	257 000	78 000	3200	200	450	0	0	82	18	8
medium tolerance	604 000	181 000	8100	470	1170	0	12	83	5	8
high tolerance	747 000	224 000	10 100	640	1490	0	44	51	6	8
*B. low border cases isolation effectiveness (20%)*										
low tolerance	216 000	66 000	2900	130	420	0	0	84	16	8
medium tolerance	557 000	167 000	7700	470	1110	0	20	75	5	8
high tolerance	689 000	206 000	9600	650	1400	6	38	50	6	8

The low- and medium-tolerance strategies respond to the number of daily reported cases whereas the high-tolerance strategy responds to the number of hospitalizations. Clearly these variables are correlated; however, their exact ratio will depend on the epidemic growth rate and lag from symptom onset to hospital admission, the age distribution of infections, the case ascertainment rate and vaccine effectiveness, all of which can vary either dynamically within the model or between different model scenarios. The average number of hospital beds occupied when a given trigger point for the number of cases is met can be calculated as a model output and is reported in electronic supplementary material, table S4.

### 3.2. Effect of border cases

Increasing the number of border cases entering the community (either as a result of an increase in travel volume or an increase in prevalence of infection in arrivals) leads to more infections, hospitalizations and deaths, although the relative impact of border cases depends on the response strategy (table 3, A1–A4; electronic supplementary material, figures S2a and S3). The number of border cases has little effect on the time spent in red or emergency setting under a low-, medium- or high-tolerance response because they are few relative to the number of community cases. For this reason, we have tested an additional ‘very low-tolerance’ scenario, with lower triggers to switch between traffic lights (table 1). Border cases make a significant difference under a very low-tolerance response, or when the number of initial community cases is zero (all cases originating at the border). This is because the very low-tolerance scenarios aim to keep community prevalence very low by quickly moving up to stricter traffic light settings (red and emergency), so the additional border cases entering the community account for a significant proportion of the total active cases.

A simple toy model can help understand the impact of border cases on the need for more stringent interventions to control community transmission. Suppose there is a predefined tolerance i* for incidence of new community infections per unit time and that public interventions switch between low and high stringency, associated with effective reproduction number *R*_1_ > 1 and *R*_2_ < 1 respectively, when incidence is below or above i*. A simple calculation based on exponential growth and decay trajectories (see ‘Derivation of toy border model’ in electronic supplementary material for details) shows that the proportion of time *p*_int_ spent under stringent interventions is

$$p_{\mathrm{int}}=\frac{R_{1}-1+b/i^{*}}{R_{1}-R_{2}},$$

where *b* is the number of border cases per unit time. This is a highly simplified model that ignores changes in *R*_eff_ due to other variables and is not suitable for quantitative estimates of the amount of time strict interventions are needed. However, it reveals the qualitative effect of key model parameters. Specifically, the relevant quantity measuring the impact of border cases is the ratio *b*/i*, i.e. the number of border cases relative to the acceptable number of infections in the community. This suggests that a higher level of tolerance for COVID-19 in the community naturally goes hand-in-hand with higher tolerance for cases at the border: if *b* is much smaller than i* then *b* could be increased with minimal impact; on the other hand if *b* is greater than (1 − *R*_2_)i* then stringent interventions will be insufficient to reduce community cases because there will still be a high number of border cases entering the community. During 2021, *R*_eff_ has mostly been greater than 0.6 in most high-income countries with ongoing epidemics, even during periods of declining cases [11]. This suggests that, to maintain control of the epidemic, given the constraints on intervention efficacy, an absolute maximum number of border cases per day would be 40% of the tolerable number of new daily infections in the community (this would mean spending 100% of the time under the more stringent setting just to keep community cases constant over time).

More generally, the equation above shows that if the switch between low and high stringency has a big impact on *R*_eff_ (i.e. *R*_1_ − *R*_2_ is large) then the relative effect of border cases *b* is small. However, if the impact of switching public health interventions is more subtle (i.e. *R*_1_ and *R*_2_ are both close to 1 so that *R*_1_ − *R*_2_ is small), the relative effect of border cases is greater. This means that border cases will be relatively more important if the strategy is to use the minimum control measures required to bring the epidemic back down below tolerance, as opposed to using highly stringent measures such as stay-at-home orders.

Changing the effectiveness of border case isolation is equivalent on average to a corresponding change in the number of border cases. Our model treats lower border case isolation effectiveness and low border case numbers in a similar way. For example, reducing effectiveness of border case isolation by a factor of 4 from 80% to 20% (table 3, B) has a similar effect as increasing the number of border cases by a factor of 4 from 5000 to 20 000 (table 3, A2).

## 4. Discussion

The main purpose of this study was to use an age-structured stochastic branching model for transmission and control of SARS-CoV-2 to better understand the effect of Aotearoa New Zealand's COVID-19 Protection Framework on the spread and impact of the Delta variant, and to provide a basis for policy advice on community control measures and border restrictions. This work pre-dates the emergence of the Omicron variant of SARS-CoV-2 and its introduction to Aotearoa New Zealand, which has necessitated a change in strategy.

We found that moderate public health measures, such as those required at the red setting of the COVID-19 Protection Framework will certainly reduce case numbers and hospitalizations. Yet, our modelling suggests that we may need to retain an emergency setting (lockdown, stay-at-home orders) to flatten the curve and avoid overwhelming the healthcare system under any of the three outbreak management scenarios investigated here.

We compared three outbreak management strategies with different levels of tolerance for COVID-19 in the community. The two lower tolerance levels used case numbers to trigger the change in traffic light, whereas the high tolerance used hospital occupancy as a trigger. The latter is a lagged indicator which depends on the vaccination status and the age distribution of cases. Using hospital occupancy as the only trigger results in a delay between the switch in traffic lights and the reduction in hospital beds occupied, whereas using case numbers as the only trigger does not take into consideration who is getting infected and what proportion of cases are in the higher risk groups which contribute the most to the health burden. There is a trade-off between health outcomes and the imposition of restrictions to control transmission. For example, under the baseline scenario and with a medium- or high-tolerance outbreak management response, there were approximately 165 000 cases and 7600 hospitalizations (peaking at 480 hospital beds occupied) over a 1-year simulation of the model, with over 70% of the time (9 months) in the red traffic light setting and 5% of the time (approx. 18 days) in the emergency setting. A low-tolerance response reduced the health burden by a factor of three, but almost tripled the time spent in the emergency setting. These low-tolerance response scenarios reflected the ‘go hard, go early’ lockdown policy strategies adopted in both New Zealand and Australian jurisdictions at the beginning of the COVID-19 pandemic in 2020 [12], which in New Zealand's case have resulted in the successful elimination of the virus from the community shortly after these public health policies were put in place. Deciding between these strategies cannot be done based on modelling alone, but should be considered alongside other measures of economic, social and health impacts (e.g. job losses, consumer spending, disrupted education impacts for mental health, rates of domestic violence). Particular attention should be given to identifying vulnerable groups who may experience inequitable impacts, for example, Māori and Pacific people [13,14], those working in customer-facing roles or precarious employment, people who are immunocompromised or have other comorbidities, and communities with high rates of crowded and inadequate housing [15–17]. Nevertheless, our model provides a rigorous framework from which to approach these trade-offs.

Case detection, case isolation and contact tracing are all essential components of the COVID-19 Protection Framework, and our findings surrounding the effects of low case tolerance strategies are sensitive to the assumptions made around our model parameters. Our sensitivity analysis showed that reducing the effectiveness of case detection and case isolation in the model led to more infections, hospitalizations and deaths under the three response strategies. Reducing the effectiveness of case detection and case isolation increased the time spent in emergency under a low-tolerance response (one extra month) relative to our baseline scenario, but had less of a profound effect on the time spent in emergency for the medium- and high-tolerance response. Increasing the contact tracing capacity from 100 to 250 cases per day had almost no effect on health outcomes or time spent in emergency setting under a medium- and high-tolerance response, because the number of cases quickly exceeded the daily contact tracing capacity. Outcomes are more sensitive to case isolation and contact tracing effectiveness under a low-tolerance strategy. This suggests that, if the strategy is to suppress the virus to very low levels, a highly effective TTIQ system is essential to minimize the need for stringent control restrictions.

As Aotearoa New Zealand opens its border to international arrivals, it is inevitable that infection importations will occur. We compared scenarios for different quarantine effectiveness for international arrivals and different numbers of border cases based on pre-COVID-19 traveller volumes. The tolerable number of cases in the community and the stringency of the measures taken to control the epidemic are the dominant determinant of the consequences of importation on local epidemic dynamics. Border cases led to more infections, hospitalizations and deaths over the year, but had a low impact on the time spent in red or emergency setting under the medium and high response scenario. A simple toy model suggested that border cases will be more important if the strategy is to use the minimum control measures required to bring the epidemic back down below tolerance, as opposed to using highly stringent measures such as stay-at-home orders. However, our model suggests that the impact of high border cases is negligible in scenarios of widespread community transmission. This result comes with the caveat that our model does not include the possible appearance of new high-transmission variants, which would enter the community through border cases and would drastically increase health outcomes [18].

Like any other modelling, this age-structured stochastic branching model contains a number of simplifications, limitations and sources of uncertainty. Firstly, the model ignores important sources of geographical and socioeconomic heterogeneities that could affect rates of transmission. For example, communities with large and crowded household and high rates of comorbidities, as well as people working in customer-facing jobs, can have higher transmission rates. Model results show the national picture for the number of cases and clinical outcomes, but these are likely to be unevenly distributed across subregions and subpopulations. Communities with low vaccination rates, high comorbidity rates and that are poorly served by healthcare systems, such as Māori and Pasifika, could be more affected by the virus. Secondly, the model does not take into account any behavioural changes that may arise dynamically as a result of the epidemic. These may change the effectiveness of interventions such as contact tracing, quarantine or prolonged restrictions. Thirdly, as shown by our sensitivity analysis on the model parameters, the number of infections, hospitalizations and deaths output by the model were most dependent on the assumptions made around (i) vaccination coverage and vaccine effectiveness against infections and onwards transmission, (ii) the effectiveness of public health and social measures effectiveness in reducing transmission of COVID-19, and (iii) the absence of new COVID variants. Although the effectiveness of the vaccine against severe disease from the Delta variant is well established, the rates of breakthrough infections and any subsequent rates of transmission in vaccinated individuals are less certain. In line with existing research [18–20], higher vaccination coverage would result in lower health outcome, while lower effectiveness against infections and onward transmission would lead to a significant increase in cases and hospitalizations numbers. In addition, the model considers immunity from the vaccine or from previous infection to remain constant over time and ignores reinfections. However, evidence suggests that immunity wanes over time [21], which would cause people that were vaccinated earlier to be at higher risk of infection as time goes on. Fourthly, the model ignores the effects of seasonality in transmission and demand on the healthcare system, population dynamics (births, deaths, ageing and migration). These factors will introduce additional complexities in keeping case numbers and mortality low, and hospitalization numbers to a manageable level. And fifthly, our model assumes a time-constant test sensitivity, independent of incubation period, whereas existing research [22] has shown a possible double effect of incubation period on transmissibility and on the delay to case ascertainment.

The response strategies we have modelled are simplifications of a real-time decision-making strategy. Other models have explored more complex and optimized combinations of triggers to switch between alert levels [23]. This work is intended to help policymakers understand the trade-offs between alternative policy options at a broad level, and is not a quantitative prediction of outcomes or a decision-making algorithm. An operational response will also need to be responsive to other variables including details of the local public health response, regional variations, transmission in high-risk groups, changes in epidemiological conditions and health system capacity.

This work pre-dates the emergence of the Omicron variant and its introduction to New Zealand. Since its arrival, the model presented here has been updated by adjusting the reproduction number, generation time, incubation period, vaccine effectiveness, hospitalization and fatality rates, and length of hospital stay, to reflect Omicron's higher infectiousness and lower severity. The model was also updated by introducing a dynamic vaccination coverage, waning of vaccine-derived and infection-derived immunity, and time-dependent behavioural changes. All these changes and the corresponding results were described in [24]. In addition, a later model update allowed us to infer the time-varying transmission coefficient from real-life case data using an approximate Bayesian computation approach [25].

## Data Availability

All data presented in this manuscript was produced using the public available code found in [26]. Model code to reproduce results and graphs: https://github.com/Giorgia93/COVID19_TrafficLights_BPMmodel.git. The data are provided in electronic supplementary material [27].
